# Correction: Development of a Multi-Biomarker Disease Activity Test for Rheumatoid Arthritis

**DOI:** 10.1371/journal.pone.0099812

**Published:** 2014-06-06

**Authors:** The PLOS ONE Staff

## Notice of Republication

This article was republished on May 19, 2014, to correct errors in equations that were introduced during the typesetting process. Please download this article again to view the correct version. The originally published, uncorrected article and the republished, corrected article are provided here for reference. The publisher apologizes for these errors.

## Supporting Information

File S1 **Originally published, uncorrected article.**
(PDF)

File S2 **Republished corrected article.**
(PDF)

## References

[1] Centola M, Cavet G, Shen Y, Ramanujan S, Knowlton N, et al (2013) Development of a Multi-Biomarker Disease Activity Test for Rheumatoid Arthritis. PLoS ONE 8(4): e60635 doi:10.1371/journal.pone.0060635 23585841 10.1371/journal.pone.0060635 PMC3621826

