# Supplementary material for: Correction: Development of a Multi-Biomarker Disease Activity Test for Rheumatoid Arthritis
Source: PLoS One. 2014 Jun 6;9(6):e99812. doi: 10.1371/journal.pone.0099812 (PMC4048285; doi:10.1371/journal.pone.0099812)
Supplement: File S1 — Originally published, uncorrected article. (PDF) [file pone.0099812.s001.pdf]

Development of a Multi-Biomarker Disease Activity Test for Rheumatoid Arthritis

Michael Centola[1]¶, Guy Cavet[2]*¶, Yijing Shen[3], Saroja Ramanujan[2], Nicholas Knowlton[4], Kathryn A. Swan[2], Mary Turner[1], Chris Sutton[1], Dustin R. Smith[1], Douglas J. Haney[2], David Chernoff[5], Lyndal K. Hesterberg[6], John P. Carulli[7], Peter C. Taylor[8], Nancy A. Shadick[9], Michael E. Weinblatt[9], Jeffrey R. Curtis[10]

1 Arthritis and Immunology, Oklahoma Medical Research Foundation, Oklahoma City, Oklahoma, United States of America, **2** Department of Informatics, Crescendo Bioscience Inc., South San Francisco, California, United States of America, **3** Department of Biostatistics and Bioinformatics, Crescendo Bioscience, Inc., South San Francisco, California, United States of America, **4** Biomarker & Proteomic Core Facility, Oklahoma Medical Research Foundation, Oklahoma City, Oklahoma, United States of America, **5** Department of Medicine, Crescendo Bioscience, Inc., South San Francisco, California, United States of America, **6** Department of Development, Crescendo Bioscience, Inc., South San Francisco, California, United States of America, **7** Genetics and Genomics Group, Biogen Idec, Cambridge, Massachusetts, United States of America, **8** Kennedy Institute of Rheumatology, University of Oxford, Oxford, United Kingdom, **9** Division of Rheumatology, Immunology and Allergy, Brigham and Women's Hospital, Boston, Massachusetts, United States of America, **10** Division of Clinical Immunology and Rheumatology, University of Alabama at Birmingham, Birmingham, Alabama, United States of America

Abstract

Background: Disease activity measurement is a key component of rheumatoid arthritis (RA) management. Biomarkers that capture the complex and heterogeneous biology of RA have the potential to complement clinical disease activity assessment.

Objectives: To develop a multi-biomarker disease activity (MBDA) test for rheumatoid arthritis.

Methods: Candidate serum protein biomarkers were selected from extensive literature screens, bioinformatics databases, mRNA expression and protein microarray data. Quantitative assays were identified and optimized for measuring candidate biomarkers in RA patient sera. Biomarkers with qualifying assays were prioritized in a series of studies based on their correlations to RA clinical disease activity (e.g. the Disease Activity Score 28-C-Reactive Protein [DAS28-CRP], a validated metric commonly used in clinical trials) and their contributions to multivariate models. Prioritized biomarkers were used to train an algorithm to measure disease activity, assessed by correlation to DAS and area under the receiver operating characteristic curve for classification of low vs. moderate/high disease activity. The effect of comorbidities on the MBDA score was evaluated using linear models with adjustment for multiple hypothesis testing.

Results: 130 candidate biomarkers were tested in feasibility studies and 25 were selected for algorithm training. Multi-biomarker statistical models outperformed individual biomarkers at estimating disease activity. Biomarker-based scores were significantly correlated with DAS28-CRP and could discriminate patients with low vs. moderate/high clinical disease activity. Such scores were also able to track changes in DAS28-CRP and were significantly associated with both joint inflammation measured by ultrasound and damage progression measured by radiography. The final MBDA algorithm uses 12 biomarkers to generate an MBDA score between 1 and 100. No significant effects on the MBDA score were found for common comorbidities.

Conclusion: We followed a stepwise approach to develop a quantitative serum-based measure of RA disease activity, based on 12-biomarkers, which was consistently associated with clinical disease activity levels.

Citation: Centola M, Cavet G, Shen Y, Ramanujan S, Knowlton N, et al. (2013) Development of a Multi-Biomarker Disease Activity Test for Rheumatoid Arthritis. PLoS ONE 8(4): e60635. doi:10.1371/journal.pone.0060635

Editor: Oliver Frey, University Hospital Jena, Germany

Received August 12, 2012; **Accepted** March 1, 2013; **Published** April 9, 2013

Copyright: © 2013 Centola et al. This is an open-access article distributed under the terms of the Creative Commons Attribution License, which permits unrestricted use, distribution, and reproduction in any medium, provided the original author and source are credited.

Funding: This work was partly supported by Crescendo Bioscience and Biogen Idec. Crescendo Bioscience was partly responsible for study design, data collection, data analysis and preparation of the manuscript. No additional external funding received for this study.

Competing Interests: The authors have the following interests. This work was partly supported by Crescendo Bioscience and Biogen Idec. GC, YS, SR, KS, DS, DH, LH: employees of Crescendo Bioscience; MC, NK, CS, DC, PT, MW, JC: consultants to Crescendo Bioscience; GC, YS, SR, KS, DS, DH, LH, MC, CS, DC: stock options in Crescendo Bioscience. GC, MC, NK, YS: inventors on US patent application #20110137851 (''Biomarkers and Methods for Measuring and Monitoring Inflammatory Disease Activity'') based on this work. JPC is employed by Biogen Idec. This paper describes the development of a diagnostics technology currently marketed by Crescendo Bioscience. There are no further patents, products in development or marketed products to declare. This does not alter the authors' adherence to all the PLOS ONE policies on sharing data and materials, as detailed online in the guide for authors.

* E-mail: glcavet@gmail.com

¶ These authors contributed equally to this work.

Introduction

RA is a common, chronic, idiopathic autoimmune disease with over 1.3 million people diagnosed in the US and over 4 million worldwide. RA is characterized by synovitis, inflammatory joint fluid, degradation of articular cartilage, erosion of the marginal bone, and systemic immune and inflammatory manifestations. Despite recent advances in treatment including the introduction of potent biologic agents, substantial disease activity persists in many patients, with accompanying progressive bone and soft tissue damage, extra-articular consequences, disability, and increased mortality.

Several studies, such as TICORA, CAMERA, BeSt, and FinRACO, have demonstrated improved outcomes with tight control of disease activity, a strategy employing frequent disease activity measurement and treatment adjustment to reach a specific target disease activity level [1–3]. Treat to Target guidelines codify these results into specific recommendations for optimal care including frequent disease activity monitoring for all patients [4]. ACR guidelines also recommend regular disease activity testing [5]. However there is no current gold standard for disease activity assessment in RA. Multiple measures are used, each with varying strengths and weaknesses, such that no single 'best' measure of disease activity could be recommended in U.S. or international RA guidelines.

Current disease activity indices are typically composite scores that can include physician assessment of symptoms, patient reported measures, and laboratory measurements. The Disease Activity Score (DAS), the Simplified Disease Activity Index (SDAI) and the Clinical Disease Activity Index (CDAI), for example, rely on joint counts, patient self-assessment and (with the exception of CDAI) laboratory tests, while the Routine Assessment of Patient Index Data-3 is based solely on PROs [6–9]. Although physician evaluation and patient self-reporting are critical components of patient assessment and management, they are influenced by intra- and inter-assessor variability and can be confounded by comorbidities or accumulated joint damage resulting from long-standing disease [10–13].

Protein biomarkers can provide complementary, objective, and reliable measurements reflecting underlying pathophysiological processes. Erythrocyte sedimentation rate (ESR) and CRP measurements are currently incorporated into clinical disease activity measures, including the DAS and SDAI. However, these biomarkers are non-specific indicators of inflammation that can be elevated due to age, anemia and the presence of immunoglobulins, and that can be unexpectedly low or even normal in patients with active disease, possibly due to underlying genetics [14–16]. Therefore, ESR and CRP measurement may not be useful in all RA patients, and other biomarkers may provide important information about disease state. Previous research studies have reported that other protein biomarkers implicated in the pathophysiology of joint disease, such as vascular endothelial growth factor-A (VEGF-A) and matrix metalloproteinase 3 (MMP3), are also correlated with disease activity [17–20]. We hypothesized that measurement of multiple serum protein biomarkers combined into a single score could quantitatively and objectively characterize RA disease activity and enhance current disease activity assessment. Periodic monitoring of this score could complement existing approaches to patient care, facilitating quantitative tracking of patient status and treatment impact and supporting management of difficult cases such as patients with comorbidities or conflicting physician vs. patient assessment. We applied a multi-step development process using multiple diverse cohorts to prioritize biomarkers of disease activity and develop such a multi-biomarker disease activity (MBDA) score for the assessment of RA disease activity. This score has subsequently been tested and validated in additional patients [21,22].

Methods

Ethics Statement

Clinical studies used as the source of biomarker samples were approved by institutional review board (Partners Institutional Review Board for BRASS, Oklahoma Medical Research Foundation Institutional Review Board for the Oklahoma City cohort, and Quorum Institutional Review Board for InFoRM) and all patients gave written informed consent.

Overview

A multi-stage approach to biomarker discovery and algorithm development was used (Table 1). In Stage 1, Screening, candidate biomarkers were identified and corresponding assays were selected and optimized. Stage 2, Feasibility, involved two parts: Stage 2A included four studies to assess and prioritize biomarkers based on their relationships to clinical disease activity; Stage 2B was a pilot imaging study to verify that multi-biomarker disease activity scores could capture critical aspects of disease activity. Stage 3, Test Development, involved further assay optimization, biomarker selection, and the training and selection of the final MBDA algorithm. Once the algorithm was finalized, the impact of comorbidities on the MBDA score was assessed.

Patient Cohorts & Samples

Biomarkers were assayed from stored serum samples obtained from patients from multiple clinical studies/cohorts. Serum was collected in standard Serum Separator Tubes in accordance with manufacturer's instructions and frozen at −80 Celsius within 72 hours. Material was maintained between 2 and 8 degrees Celsius until freezing except in the BRASS cohort, for which SST tubes were shipped at ambient temperature prior to separation of serum by centrifugation. In all studies except the Stage 2B Pilot Imaging study, observational cohorts were used. The objective was to evaluate the intended use population for the MBDA test: diverse patients representative of the RA population in the United States and Western Europe, treated according to current practice norms. The reasons for using multiple observational cohorts were 1) to ensure that only biomarkers that behaved consistently across different patient populations would be selected for use in the final MBDA algorithm, and 2) to access sufficiently large numbers of patients for adequate statistical power. In the Stage 2B Pilot Imaging study the objective was to examine the relationship between disease activity biomarkers and disease measures based on joint imaging, and the cohort was selected because of the availability of high-quality ultrasound and X-ray image data. In all studies the determination of clinical disease activity was carried out using standard methods (refs) and the assessment was carried out without knowledge of biomarker concentrations (which were determined later).

All patients fulfilled at least 4 of 7 of the 1987 revised ACR criteria for RA [23]. Exclusion criteria applicable to all source cohorts were: oral (>10 mg/day) or parenteral (any) corticosteroid use within the last 4 weeks; women who were pregnant, nursing, or planning pregnancy within 6 months of study enrollment; signs or symptoms of severe, progressive or uncontrolled renal, hepatic, hematologic, gastrointestinal, endocrine, pulmonary, cardiac, neurologic, or cerebral disease; concomitant diagnosis or history of congestive heart failure; a known history of a demyelinating

Table 1. Staged approach used in biomarker discovery and prioritization and algorithm development.

Stage		Study	Objectives	Biomarkers	Patients	Samples
SCREENING	1	-	Candidate marker identification; Initial assay optimization	130*	20	20
FEASIBILITY	2A	Study I	Prioritization	113	128	128
FEASIBILITY	2A	Study II	Prioritization	75	320	320
FEASIBILITY	2A	Study III	Prioritization	65	85	255
FEASIBILITY	2A	Study IV	New marker evaluation & Prioritization	16	119†	119†
FEASIBILITY	2B	Pilot Imaging	Assessment of capabilities of biomarker-based disease activity scores	>25‡	24	107
DEVELOPMENT	3	Training	Analytical validation; Development & testing of candidate algorithms	25	708	708

*130 biomarkers had adequate measurability to advance to studies of clinical disease activity.
†Patients and samples in Study IV represent a subset of those evaluated in Study II.
‡In addition to the 25 biomarkers that were subsequently advanced to model development (Stage 3), this study also examined other serum biomarkers of potential interest to prediction of structural damage progression, some of which overlapped with biomarkers considered for disease activity prediction.
doi:10.1371/journal.pone.0060635.t001

disease; any known malignancy currently or within the previous 5 years (with the exception of basal cell or squamous cell carcinoma of the skin that had been fully excised with no evidence of recurrence); seropositivity for HIV; active infection or active substance abuse. See Table 2 for cohort characteristics.

Feasibility studies. Serum samples examined in Feasibility Studies I–IV were derived from the Oklahoma City cohort, an observational study of patients seen at community clinics located in and around Oklahoma City, OK, and from the Brigham and Woman's Rheumatoid Arthritis Sequential Study (BRASS) Registry [24,25], an observational cohort study of patients seen at the Brigham and Women's Hospital in Boston, MA. For Study I, single visit samples were obtained from 128 patients with RA from the Oklahoma City cohort. For Study II, single visit samples were obtained from an additional 140 patients from the Oklahoma City cohort and 180 patients from the BRASS registry. For Study III, serum samples at baseline, 1 year, and 3 years were obtained from each of 85 patients in the BRASS registry, in order to evaluate the utility of longitudinal disease activity data. For Study IV, single visit samples were analyzed from 119 patients among the 140 Oklahoma City cohort patients from Study II for whom sufficient residual sample volume was available, in order to examine new candidate biomarkers.

Pilot ImagingStudy. Serum samples at multiple time-points (0, 6, 18, 52, 110 weeks) were obtained from 24 patients followed in a 2-year blinded study in the UK comparing methotrexate+in-

Table 2. Patient characteristics in Feasibility (Stage 2) studies.

	Study I	Study II	Study III	Study IV	PoC Study
Number of patients/samples	128/128	320/320	85/255*	119/119**	24/107*
Female, %	82	80	91	77	75
CCP+, %	63	62	62	61	n/a
RF+, %	83	83	64	97	n/a
Smoker, %	n/a	13	4	22	n/a
Methotrexate, %	53	61	48	64	100
Non-biologic DMARDs, %	69	76	64	81	100
Biologics, %	65	53	43	50	50
Corticosteroids, %	24	27	27	33	n/a
Age, mean±SD (min,max)	60±13	59±14	59±13	60±14	56±13
DAS28-CRP, median (IQR)	5.8 (4.7–6.5)	4.0 (2.9–5.3)	3.8 (2.7–5.0)	5.2 (4.1–6.2)	3.3 (2.2–4.4)
TJC28, median (IQR)	12 (4.8–20)	2.0 (0–8.3)	7.0 (2.0–14)	8.0 (3.0–15)	3.0 (0.0–8.0)
SJC28, median (IQR)	16 (12–21)	6.5 (2–13)	2.0 (0.0–10)	14 (8.0–20)	4.0 (1.0–7.0)
CRP mg/L, median (IQR)	14 (4.0–32)	14 (5.1–45)	14 (4.0–47)	18 (6.9–47)	25 (7.6–70)
PG, median (IQR)	5.0 (2.9–7.0)	2.5 (1.0–5.0)	3.0 (1.0–5.0)	5.0 (2.0–6.5)	n/a

DMARD, disease-modifying anti-rheumatic drug; IQR, inter-quartile range.
*For studies with multiple samples per patient, sex, age, and serological status (when available) statistics are based on unique patients. Other statistics are based on all samples.
**All studies used independent patients and samples, except Study IV, which used a subset of Study II samples.
doi:10.1371/journal.pone.0060635.t002

fliximab combined therapy with methotrexate monotherapy in aggressive early RA [26,27]. Patients were evaluated with ultrasound (US) at 0, 18, 54 and 110 weeks and scored for synovial thickening (ST) and for vascularity by power Doppler area (PDA). Details of imaging and scoring have been previously described [26]. Briefly, each MCP was scored for ST using grayscale images on a 0–5 scale, and the overall ST score was the total of the scores for the individual joints. The numbers of pixels showing Doppler signal were summed across the 10 MCPs to give the overall PDA score. Radiography was performed at 0, 54, and 110 weeks and used to determine van der Heijde-modified total Sharp scores (TSS).

Algorithm training. Single samples were obtained from each of a total of 703 patients from the BRASS cohort and from the Index for Rheumatoid Arthritis Measurement (InFoRM) study [28], a multicenter North American observational study conducted by Crescendo Bioscience, South San Francisco, SF. Five hundred and twelve patients from InFoRM were selected (from ~1300 total) to be representative of the overall disease activity distribution in the study and also of the broader North American RA population from which the InFoRM cohort was drawn. An additional 29 InFoRM patients were selected to enrich for high and low disease activity. One hundred and sixty-seven patients were selected from the BRASS registry to capture the full range of disease activity, enriching for very low and high disease activity (although 5 were subsequently excluded from analysis due to incomplete clinical data). Representation of low and high disease activity patients was enriched to increase the power to detect associations between biomarkers and clinical disease activity, and to ensure that the MBDA algorithm worked across the full disease activity range. All patients used were independent from those assessed in the prioritization studies and pilot imaging study listed above. The 703 samples were used to further prioritize the individual biomarkers based on their associations with clinical measures of disease activity. A subset of these patients was used for the final fitting of the statistical models used in the MBDA algorithm. In order to fit models optimally, it was important to have substantial variation in disease activity levels; therefore, the subset of 249 samples was selected to have similar numbers of patients in low, moderate and high disease activity. The performance of the most promising models from training was evaluated in an independent 70-sample subset of the 703 samples, selected to have similar disease activity composition to the final 249-sample training set. The best models were further evaluated in patients from the computer-assisted management in early rheumatoid arthritis (CAMERA) study [21].

Comorbidities study. Samples from the 512 representative InFoRM patients used in algorithm training were analyzed to evaluate the impact of common comorbidities on the MBDA score. The presence or absence of comorbidities was recorded by study investigators based on their individual clinical knowledge. The case report forms did not specify diagnostic or classification criteria for diseases other than RA.

Candidate Biomarker Selection

Literature analysis. Candidate biomarkers previously reported to be related to RA disease activity or underlying processes were identified through manual review of scientific articles and bioinformatics databases of findings extracted from the literature. Manual literature searches were conducted using PubMed (http://www.ncbi.nlm.nih.gov/pubmed/). Bioinformatics approaches employed IRIDESCENT [29] and Ingenuity Pathways Analysis (Ingenuity Systems, Redwood City, CA).

Assays & Optimization

Detection of anti-cyclic citrullinated peptide (CCP) and rheumatoid factor (RF) antibody activity. For the Oklahoma cohort samples, anti-CCP was measured using a commercially available ELISA kit (Quanta Lite CCP 3.1 IgG/IgA Kit, INOVA Diagnostics Inc., San Diego, CA) and RF was measured with the EL-RF/3 kit (Theratest Laboratories, Lombard IL). In the BRASS and InFoRM cohort samples both RF and anti-CCP were measured using Cobas analyzers (Roche Diagnostics, Indianapolis, IN) according to manufacturer's instructions.

Candidate biomarker assays. All assays were run in 96-well plates with 8 point standard curves (7 standards and a blank). Both standards and patient samples were run in duplicate in adjacent wells on the same plate in all studies. A single lot of each assay reagent was used in each study wherever possible to minimize plate to plate variation. Pools of commercially available human sera (Bioreclamation Inc., Nassau, NY) from rheumatoid arthritis patients, osteoarthritis patients, systemic lupus erythematosus patients and unaffected controls were run as process controls on each plate. Luminex-based assays were run on either a Luminex 100 or Luminex 200 device and analyzed using either Bioplex curve fitting (Biorad Inc. Hercules, CA) and 4-parameter logistic (4PL) curve fitting with Power Law Variance weighting or Xponent software (Luminex Inc. Austin TX) with 4PLcurve fitting and $1/y^2$ weighting. Meso Scale Discovery (MSD) assays were evaluated using a Sector Imager 6000 device (Meso Scale Discovery, Gaithersburg, MD) using MSD Discovery Workbench software with 4 parameter logistic curve fitting and $1/y^2$ weighting. ELISA-based assays were evaluated using a BioTek ELx800 plate reader (BioTek Inc. Winooski, VT) and TERIS software with 4 parameter logistic curve fitting and 1/y weighting. Sample and standard/calibrator dilutions were adjusted such that marker levels were best positioned in the linear portions of standard curves. For Stages 1 and 2, serum from 20 RA patients with a representative distribution of DAS scores was used to optimize assay sensitivity and dynamic range, to best map RA patient protein measurements onto the linear portion of the standard curve, and to minimize serum volume requirements. Tested assays are listed in Table S1, which also indicates how assays were multiplexed and the suppliers of assay materials including standards. Final sample dilutions, assay precision and measurability limits are reported in Table S2. For Stage 3 (Development), assays were optimized and characterized as described previously so as to be highly consistent across studies and suitable for use in a clinical diagnostic test [30]. Details of assay multiplexing, sample dilutions, limits and sources of standards are given in Table S3.

Heterophilic antibody and RF interference. Assays sensitive to heterophilic antibody activity were identified by evaluating whether blocking reagent (Heteroblock, Omega Biologicals, Bozeman, MT) reduced signal in 5 samples with high RF titers but not in 5 samples with low RF. If an assay showed evidence of interference, the optimal Heteroblock concentration for that assay was determined by identifying the minimum concentration that suppressed spurious signal in high RF samples, as previously described [31]. Heteroblock was then added to all subsequent plates for that assay.

Biomarker data quality control. Quality control of each assay plate was conducted by monitoring the performance of the process controls (the serum pools diluted alongside the samples on each plate) and, for Stage 3, also of run controls (made from standard material). In stages 1 and 2, consistency of control recovery was compared to the other plates within a study and across studies in order to identify any outlier plates that warranted

repeat assay runs. When individual sample signal in a study was too low to fall on the standard curve, the concentration was set to the lowest value observed for any sample in that study. Conversely if the signal was too high to fall on the standard curve, the concentration was set to the highest value observed. In Stage 3, control tables were established for control samples with acceptability ranges being defined as ± 3 standard deviations (SD) of the expected value. If multiple run controls or process controls on a plate had observed concentrations outside the acceptability range for any given assay, the plate was flagged for review and repeated if necessary. For plates that were flagged for review, standard curves were examined (i.e. % recovery versus expected concentrations and consistency among duplicate wells) and edited if necessary to remove outlier wells. If the signal coefficient of variation (CVs) between a sample's duplicate wells was greater than 20%, the sample was flagged for review. If either a plate or sample was determined to be unacceptable, a data review team decided whether to either remove the data from the analysis or re-run if additional sample volume was available.

Statistical Analysis & Modeling

Biomarker evaluation & prioritization. In each study, biomarkers were assessed and prioritized based on univariate and multivariate association with 8 clinical measures (DAS28-CRP4, DAS28-ESR4, CDAI, SDAI, swollen joint count [SJC], tender joint count [TJC], and patient global assessment (PGA)), both in the entire study cohort and in subgroups defined by autoantibody status, sex, and therapy. Data for each study was analyzed separately. The need to adjust data for plate to plate variation was assessed for each study. Univariate associations between marker levels and clinical measures were assessed using Pearson and Spearman correlations. Statistical significance was assessed after correction for multiple testing (FDR<20%, [32]). Multivariate modeling was performed using Ordinary Least Squares (OLS) regression, Least Absolute Shrinkage and Selection Operator (LASSO) [33], and elastic net [34] modeling approaches, each with forward stepwise selection of biomarkers.

In each study, a univariate rank was calculated for each biomarker based on the number of times the marker passed statistical significance criteria in univariate analyses using different disease activity metrics and subgroups. For multivariate analysis, a priority was assigned for each biomarker for each multivariate model based on order of entry into the model (the first biomarker included in a model received a priority of 1, etc.). A score was calculated for each biomarker as the sum of the inverse of the biomarker's priority values across all models. Ranks for multivariate analysis were determined by sorting biomarkers in descending order of this score. Biomarkers not included in any model were assigned a common lowest rank. To combine ranking information from univariate and multivariate analyses, a combined score was calculated as the sum of the inverse univariate rank and the inverse multivariate rank. Combined ranks were determined by sorting the biomarkers in descending order of combined score. The combined rank was used to inform biomarker advancement from one Feasibility study to the next. At the end of Stage 2, the inverse combined ranks from all of the Stage 2 studies were summed to determine an overall score. Biomarkers were sorted in descending order of the overall score to determine a "grand rank" that summarized evidence from multiple studies. This grand rank was used, along with other criteria as described in Results, to inform advancement to algorithm training in Stage 3.

Pilot imaging analyses. The pilot imaging study included evaluation of association between disease activity levels predicted by multivariate biomarker models and US synovitis, Power Doppler area, and subsequent change in TSS, as well as the association between change in biomarker-based disease activity score and change in DAS28-CRP. Leave one out cross-validation [35] was used to generate biomarker-based disease activity scores, whereby the score for each patient were made using a model trained using DAS28-CRP data from all the other patients. Association with other measures was assessed by Spearman correlation. Cross-sectional correlation to ultrasound was determined at all time-points for which paired biomarker/US measurements were available. Longitudinal correlation to damage progression was evaluated for baseline biomarker-based disease activity scores vs. change in TSS from baseline to year 1, and for year 1 disease activity scores vs. change in TSS from year 1 to year 2. Correlation between change in biomarker-based disease activity scores and change in DAS28-CRP was evaluated for changes from baseline to year 1 and from year 1 to year 2.

Algorithm training. Multivariate methods considered in algorithm development included OLS, LASSO, elastic net, and also approaches combining the Curds & Whey (CW) multivariate response method [36] with OLS or LASSO (CW-OLS and CW-LASSO respectively). Algorithm performance was measured by Pearson correlation to DAS28-CRP and the area under the receiver operating characteristic curve (AUROC) for classifying patients into low vs. moderate to high disease activity using a DAS28-CRP cutoff of 2.67. To minimize variability of performance estimates due to unequal numbers of patients in low and moderate/high disease activity groups, the AUROC was also assessed using an alternate threshold equal to the study median DAS28-CRP. The performance of the regression methods was compared in 70/30 cross-validation (repeatedly training in a randomly selected 70% of the data and testing in the remaining 30%). The number of biomarkers in each regression model was chosen using nested 10-fold cross validation: within a training set, samples were divided into 10 equal groups, models were built using data from 9 groups, and performance was tested in the one group that was not used for model building. This process was repeated, each time with a different group set aside for independent testing, and then the performance was averaged across all models. This procedure was used for models with all possible numbers of variables, and the number of variables with the best performance was chosen. In the CW approaches, nested 10-fold cross-validation was used for each sub-model corresponding to each component of DAS to identify the optimal number of variables for that sub-model. The performance of the MBDA algorithms identified in training was evaluated by Pearson correlation to DAS28-CRP in the 70-patient test data set, and the top performing models were selected.

Comorbidities analysis. Comorbidities were characterized according to physicians' responses on InFoRM case report forms. The median MBDA score, CRP, CDAI, and DAS28-CRP were compared in subgroups of RA subjects with and without various comorbid conditions, using the ratio of the median values in the two groups. A ratio of 1.0 indicated that the value of the score was not affected by the comorbidity; ratios meaningfully higher or lower than 1.0 suggest that the comorbidity may inappropriately raise or lower the score, respectively. Multiple linear regression was used to control for age and sex. The effect of multiple testing was controlled using the false discovery rate method of Benjamini and Hochberg [32].

Statistical analysis was carried out with the R programming language (http://www.r-project.org) unless otherwise specified.

Results

Stage 1: Screening

The objective of the Screening stage was to identify proteins that could be measured in serum and that had potential to show association to RA disease activity. We focused on serum testing due to the standard use of serum in clinical rheumatology testing as well as existing literature suggesting the association of multiple serum proteins with RA disease activity [17,20,37–41]; see also Table S1. A total of 130 candidate biomarkers were selected based on comprehensive literature review, previous experiments, the availability of immunoassay components, and evaluation of measurability in serum (see Methods). These candidate biomarkers were then evaluated by examining their association with clinical disease activity measures in a series of Feasibility studies.

Stage 2: Feasibility

Patient characteristics for the 4 Feasibility studies are provided in Table 2.

Feasibility stage 2A – biomarker prioritization. In Stage 2A, four successive studies were used to prioritize and reduce the candidate biomarker list to a final set of biomarkers for algorithm development. Table S4 provides the list of biomarkers evaluated in each study, along with examples of univariate correlation results and biomarker rankings.

Study I was an initial assessment of the utility of candidate biomarkers for predicting disease activity, in order to prioritize roughly 50 biomarkers for further testing. Testing was done with 113 biomarkers with satisfactory assay performance in the Screening stage. Ninety-one different assays for 72 unique biomarkers received a rank reflecting their contribution to multivariate disease activity models. Fifty-two of these biomarkers were selected for advancement based on their combined rank from univariate and multivariate analysis.

Studies II, III, and IV were used for further refinement of the biomarker set. Seventy-five biomarkers were assessed in Study II: in addition to the 52 markers that were selected based on combined rank in Study I, another 23 were included due to strong univariate relationship to disease activity in Study I or because the assays were included on the same multiplex assay as other prioritized biomarkers. Of the 75 biomarkers tested in Study II, 65 were also analyzed in Study III (10 were eliminated due to low performance). In Study IV, we then assessed 16 new biomarkers that we had not previously tested, either because they were newly identified as candidate biomarkers or because assays had recently become available.

In Study II, we used results from multivariate analysis to review the potential value of prototype multi-biomarker models for disease activity. In cross-validation, multi-biomarker models had a mean Pearson correlation with DAS28-CRP of $r = 0.60$, compared to the highest correlation of any individual biomarker to DAS28-CRP of $r = 0.38$, observed in this study for CRP. In an AUROC analysis examining the ability of the models to classify patients into either low disease activity or moderate/high disease activity categories using the clinical cutoff of 2.67 [42], the average AUROC was 0.89 (Figure 1). Using the median DAS28-CRP as threshold the average AUROC was 0.77.

Combined univariate/multivariate biomarker ranks were determined in each of the 4 prioritization studies and guided decisions on marker advancement between studies. Because Study 4 was performed in a subset of patients previously analyzed in Study II, data on the 16 new biomarkers was combined with measurements of the previous 75 biomarkers to determine the ranks in this study.

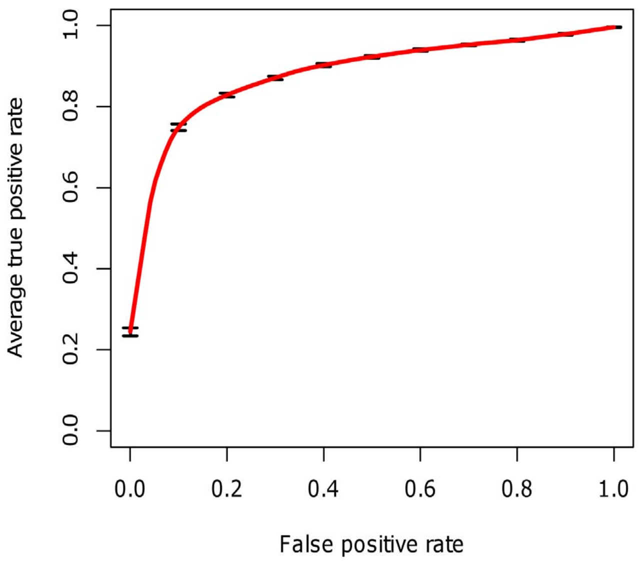

Figure 1. Receiver operating characteristic curve for biomarker-based multivariate models of disease activity in Study II. Curve shows the average true positive rate across 100 folds of cross validation. In each fold a model was trained on a randomly selected 70% of the data and performance was tested on the remaining 30%.
doi:10.1371/journal.pone.0060635.g001

The results of the 4 biomarker prioritization studies were then analyzed together to determine a final biomarker set for advancement to algorithm training. The grand ranks calculated from the integrated results of the 4 studies (see Methods) are provided in Table S4. Biomarkers with grand rank ≤24 were prioritized for advancement to training, with the following exceptions: apolipoprotein-B was eliminated due to poor assay performance, BAFF was eliminated because of evidence that the B-cell depleting therapy rituximab led directly to large increases in marker level [43], and CXCL10 and GM-CSF were eliminated because their high ranking resulted entirely from good performance in a single study. Furthermore, four lower-ranking biomarkers were selected due to their representation of key biological functions or pathways in RA, especially those implicated by other prioritized biomarkers: VCAM1 was included as complementary to ICAM1 (adhesion molecules), IL1b was included for its relation to IL1Ra (IL-1 pathway), MMP1 was included for its similarity to MMP3 (both MMPs) and CCL22 (MDC) was included to represent monocyte/macrophage biology. Thus the sequence of prioritization studies yielded a set of 24 biomarkers.

Feasibility stage 2B – pilot imaging study. In parallel with the later stages of biomarker prioritization, we assessed whether prototype multivariate models of disease activity based on serum biomarkers demonstrated various critical aspects of an effective disease activity measure, specifically: an association with clinically-assessed disease activity and changes therein; an association with imaging-based assessment of joint inflammation; and an association with subsequent radiographically assessed damage progression.

The 24 prioritized disease activity biomarkers were assessed alongside other biomarkers being evaluated for prediction of structural damage progression in samples from a 2-year study of clinical, ultrasound, and radiographic outcomes in early aggressive RA [26,27]. Using data for all the tested biomarkers, multivariate models were trained to estimate disease activity as measured by the

DAS28-CRP, and resulting biomarker-based scores were evaluated for relationships to ultrasound and radiographic measurements of joint status and damage progression. In leave one out cross-validation, the biomarker-based disease activity scores were significantly correlated with DAS28-CRP (Spearman's rho = 0.82, P<0.001, Figure 2a). The biomarker-based disease activity scores were also significantly correlated with both synovial thickening (rho = 0.46, P<0.001) and joint vascularity (rho = 0.47, P<0.001) measured by power Doppler ultrasound. Furthermore, the biomarker-based disease activity scores were significantly associated with subsequent changes in total Sharp score. For example, biomarker-based scores calculated at baseline and year 1 had a Spearman correlation with 12 month change in total Sharp score (baseline to year 1, year 1 to year 2, respectively) of 0.52 (P<0.001, Figure 2b) compared to a correlation of 0.43 (P = 0.006) between the DAS28-CRP and changes in total Sharp Score. Biomarker-based disease activity scores and subsequent 12-month change in Sharp score were also correlated when calculated separately for the two years of the study (baseline scores vs. first year change correlation = 0.44, P = 0.05; year 1 scores vs. second year change correlation = 0.61, P = 0.005). Because this study included serial measurements of disease activity by DAS28-CRP in each patient, we were able to evaluate whether the biomarker-based disease activity scores could also track changes in clinical disease activity. Indeed, changes in the biomarker-based scores were significantly correlated to changes in DAS28-CRP, with a Spearman correlation of 0.62 (P<0.001, Figure 2c).

Finally, in the process of evaluating the relationship of the different biomarkers with disease activity, it became apparent that pyridinoline, one of the biomarkers being investigated for structural damage prediction in a separate study, was strongly correlated to DAS28-CRP (rho = 0.47, P<0.001). Thus, pyridinoline was added to the marker set for Stage 3 studies, yielding a set of 25 prioritized biomarkers.

Development: Stage 3

Assay optimization. To ensure that the prioritized biomarker assays were sufficiently precise and specific for use in a clinical diagnostic test, we optimized the individual biomarker assays to function in a multiplex environment with precision across time, instruments, operators, and reagent lots [44]. Of the 25 biomarkers entering development efforts in Stage 3, seven were eliminated at various points in Stage 3 due to practical considerations: interleukin 8 (IL-8) was highly sensitive to variation in sample collection conditions (e.g. shipping at ambient temperature prior to serum separation); interleukin 1 beta (IL-1b) levels were below the limit of detection in many patient samples; interleukin 6 receptor (IL-6R) was eliminated because its levels are dramatically affected by treatment with tocilizumab (anti-IL-6R antibody); and pyridinoline, calprotectin, and apolipoproteins AI and CIII were eliminated because their assays did not meet the performance criteria required for clinical testing.

Algorithm training. In the first part of algorithm training, the entire data set of 703 patients was used to characterize the relationships between the biomarkers and disease activity, while a 249-patient subset was used for final training. Demographics of the cohorts used in algorithm training are presented in Table 3.

Various statistical approaches were evaluated for algorithm training (see Methods). A combination of "Curds and Whey" [36] and LASSO [33] approaches (CW-LASSO) yielded the best performance in cross-validation in training and algorithm testing (data not shown). LASSO modeling with forward stepwise variable selection and 10-fold cross-validation was used to select biomarkers and optimize a linear model for each DAS28-CRP component (except CRP). Then the Curds and Whey method was applied to improve upon the LASSO-derived predictions by exploiting correlations between the components, through application of a shrinkage matrix [36]. This approach improved performance of LASSO-derived joint count predictions, whereas analysis from cross-validation showed that inclusion of predicted PGA or CRP in the shrinkage matrix would have impaired performance.

The final algorithm was a 12-biomarker model for the multi-biomarker disease activity (MBDA) score. In this algorithm, 11 biomarkers (tumor necrosis factor receptor I (TNF-RI), interleukin 6 (IL-6), vascular cell adhesion molecule 1 (VCAM-1), epidermal growth factor (EGF), VEGF-A, cartilage glycoprotein 39 (YKL40), matrix metalloproteinase 1 (MMP1), MMP3, serum amyloid A (SAA), leptin, and resistin) are used for prediction of the TJC28, SJC28 and PGA, with different biomarkers and weightings used to predict each component (Figure 3). Marker concentrations were transformed to the power 1/10 to produce approximate normality and improve the robustness of the multivariate models. The component predictions are then combined with CRP in an equation analogous to that used to calculate the DAS28-CRP, and results are rounded and scaled to produce integer-valued MBDA scores in the range 1–100 (Equations 1–7). The contribution of CRP to the MBDA score is similar to its contribution to the DAS28-CRP. In the 512 InFoRM patients used in Development, CRP contributed an average of 16% of the overall score (SD 6%), and 25% of the non-constant portion of the score (SD 13%; the portion remaining when the contribution of the 0.96 constant from the DAS28-CRP formula is removed). Thresholds for MBDA-based disease activity classification are shown in Table 4; category cutoffs were determined from the corresponding DAS28-CRP values [45] using the relation specified in the MBDA algorithm: MBDA = round (DAS28-CRP*10.53+1).

DAS28-CRP and MBDA formulas:

$$DAS28-CRP=0.56\sqrt{TJC28}+0.28\sqrt{SJC28}$$
$$+0.14PG+0.36\ln(CRP+1)+0.96$$

$$MBDA=round[\max(\min((0.56\sqrt{PTJC28}+0.28\sqrt{PSJC28}$$
$$+0.14PPGA+0.36\ln(CRP+1)+0.96)$$
$$\times 10.53+1,100),1)]$$

LASSO-derived component models:

$$PTJC28^{(0)}-38.564+3.997[SAA]^{(1/10)}+17.331[IL6]^{(1/10)}$$
$$+4.665[YKL40]^{(1/10)}-15.236[EGF]^{(1/10)}+2.651[TNFRI]^{(1/10)}$$
$$+2.641[Leptin]^{(1/10)}+4.026[VEGFA]^{(1/10)}-1.470[VCAM1]^{(1/10)}$$

CW-derived improved models.

$$PSJC28=max(0.173\times PTJC28^{(0)}+0.784\times PSJC28^{(0)},0)$$

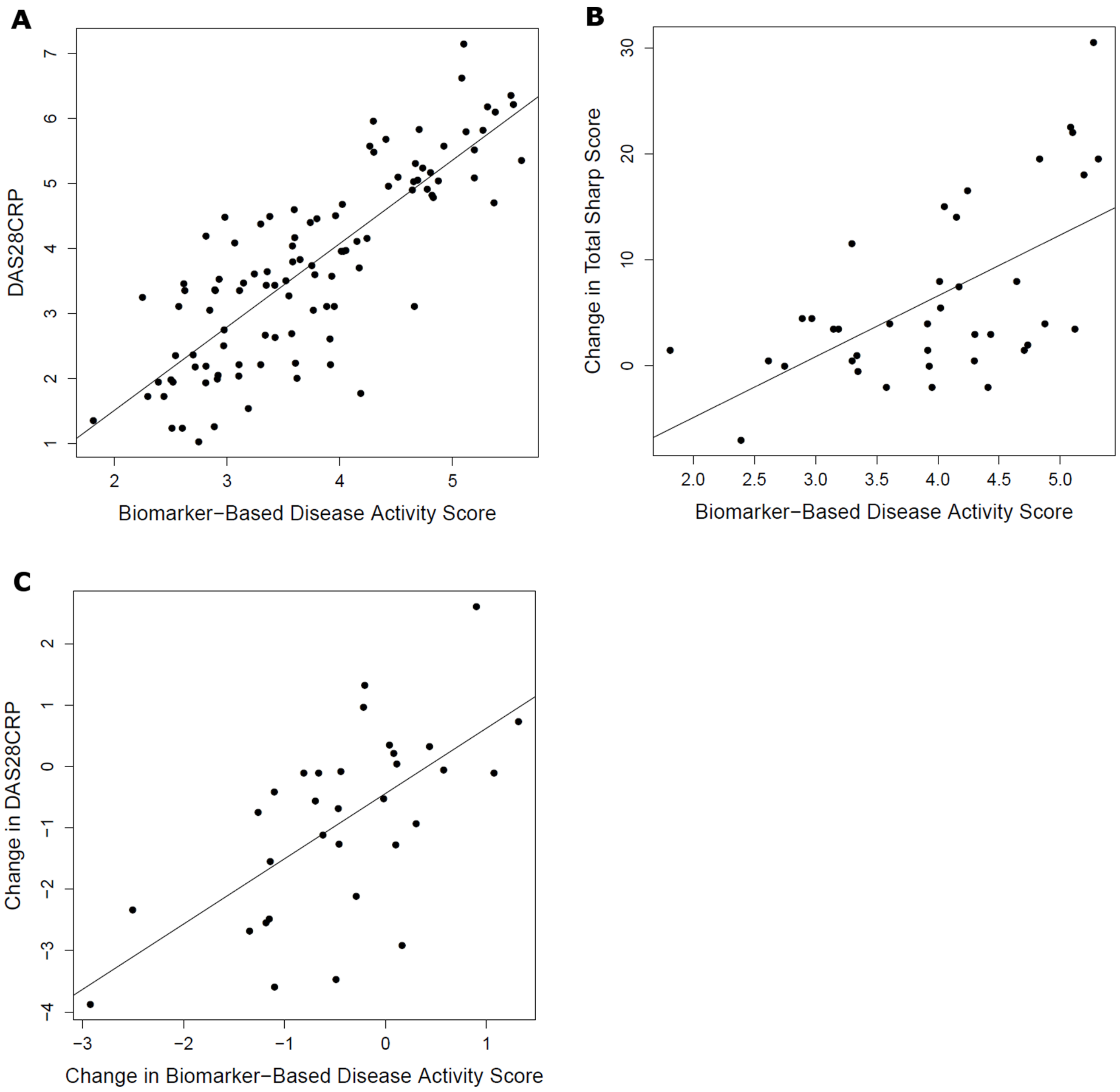

Figure 2. Multivariate biomarker-based disease activity predictions in relationship to other measurements in Pilot Imagingstudy. Prototype biomarker-based disease activity scores vs. DAS28-CRP (for all time-points with both DAS28-CRP and biomarker measurements) (a); baseline and year 1 values of biomarker-predicted disease activity vs. change in TSS from baseline to year 1 and from year 1 to year 2 (b); and change in biomarker-predicted disease activity vs. change in DAS28-CRP from baseline to year 1 and from year 1 to year 2 (c). Biomarker-based models were trained against DAS28-CRP and produce scores on a similar scale.
doi:10.1371/journal.pone.0060635.g002

PTJC28, predicted 28 tender joint count; PSJC28, predicted 28 swollen joint count; PPGA, predicted patient global assessment.

Impact of comorbidities on MBDA score. Because comorbidities could influence clinical and biomarker-based disease activity assessment, we evaluated the impact of various comorbidities on the MBDA score as well as on clinical disease activity measures (DAS28-CRP, CDAI and CRP) in the subset of 512 representative InFoRM patients that was used in algorithm development. The following comorbidities were present in 10% or more of the subjects and were assessed in this study: hypertension, osteoporotic fracture, degenerative joint disease, diabetes, and asthma. There were no statistically significant associations between any of these comorbidities and any of the measures of disease activity when adjusting for age and sex, and accounting for multiple comparisons (Table 5).

Discussion

Advances in biomarker analysis in recent years have enabled the development of multi-biomarker based diagnostics tests that are impacting patient care and outcomes in various therapeutic areas.

Table 3. Baseline characteristics of patients used for Training and Comorbidities studies.

	Training (final fitting)	Comorbidities (InFoRM 512)
Number of samples	(n = 249)	(n = 512)
% Female	75	76
% RF+	61[a]	77[b]
% anti-CCP+	58[c]	65[d]
Median age (IQR)	58 (49–67)	59 (50–68)
Median DAS28-CRP (IQR)	3.8 (1.6–6.4)	3.3 (2.3–4.7)
Median TJC (IQR)	5 (0–18)	2 (0–8)
Median SJC (IQR)	4 (0–17)	2 (0–6)
Median CRP, mg/L (IQR)	3.8 (1.3- 20.5)	4.3 (1.9–12)
Mean PG (IQR)	3.9 (1–7)	3.5 (1–5.5)
Mean MBDA (IQR)	42 (33–50)	40 (30–59)

RF and anti-CCP status was not available for all patients, evaluable patients noted:
[a]n = 198;
[b]n = 505;
[c]n = 232;
[d]n = 511.
IQR, inter-quartile range.
doi:10.1371/journal.pone.0060635.t003

Tests based on tissue and peripheral blood gene expression, and serum protein levels, for example, have been introduced for application in breast cancer, heart transplant, and type 2 diabetes, respectively [46–48]. In order to develop clinically useful tests for other clinical applications it is important to use adequately powered studies from diverse clinical cohorts, technically optimized and validated protein assays, rigorous statistical analysis and multiple sequential studies. We have applied this approach to develop a multi-biomarker disease activity (MBDA) test to aid in the assessment of RA patients.

Table 4. Disease activity category definitions for DAS28-CRP and MBDA.

Disease activity category	DAS28-CRP definition	MBDA definition
Remission	<2.3	≤ 25
Low	≤ 2.7	≤ 29
Moderate	>2.7 & ≤ 4.1	>29 & ≤ 44
High	>4.1	>44

doi:10.1371/journal.pone.0060635.t004

Testing in multiple diverse clinical cohorts was a critical aspect of the development process. Biomarker performance may vary between cohorts because of a range of factors including ethnicity, recruitment biases, practice patterns and technical differences. Patients were selected from multiple North American and European sites and studies to capture diversity in disease activity, disease duration, treatment approaches, sex, serological status and other patient characteristics. This diversity is needed to ensure that the resulting algorithm will be applicable to diverse RA patients despite the extensive clinical heterogeneity seen in RA.

Technical assay optimization and validation were essential to ensure that assay results are consistent and reproducible over time. In RA patients it was also necessary to ensure that there was no direct interference due to interaction from RA drugs, heterophilic antibodies such as RF, or other substances. Only assays with

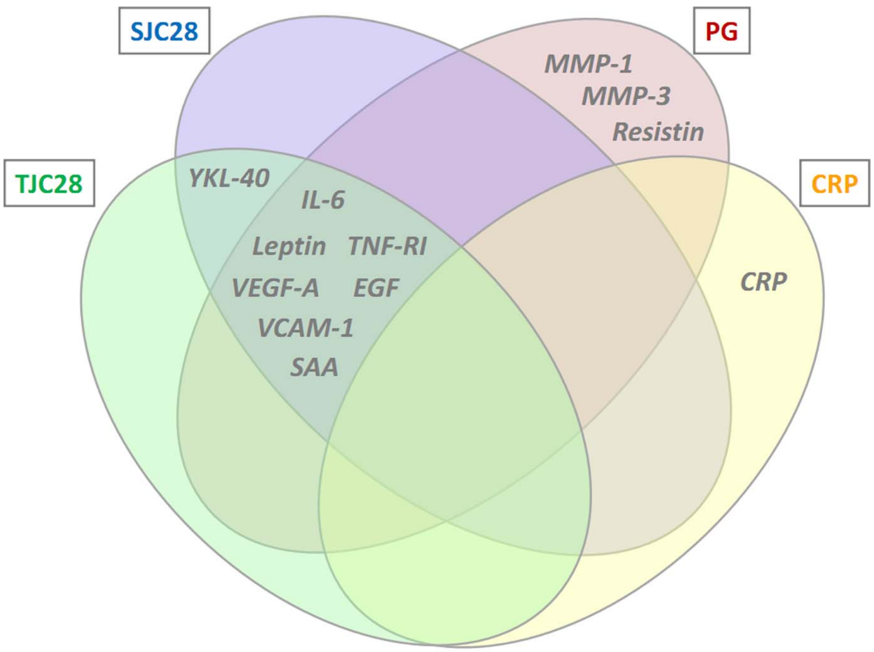

Figure 3. MBDA score algorithm. The MBDA score algorithm uses an equation analogous to that for the DAS28-CRP, with biomarkers used to predict the Swollen Joint Count (SJC28), Tender Joint Count (TJC28), and Patient Global Assessment (PG) components of the equation. The Venn diagram lists the MBDA score biomarkers used to predict each MBDA score component. YKL-40, human cartilage glycoprotein-39; IL-6, interleukin-6; SAA, serum amyloid A; EGF, epidermal growth factor; TNF-RI, tumor necrosis factor receptor 1; VEGF-A, vascular endothelial growth factor-A; MMP, matrix metalloproteinase.
doi:10.1371/journal.pone.0060635.g003

Table 5. Ratios of median disease activity measures* between RA patients with and without common comorbidities.

Comorbidity	n (%)	CRP	CDAI	DAS28-CRP	MBDA Score
Hypertension	223 (44)	0.98	1.32†	1.14†	1.05
Osteoarthritis‡	172 (34)	0.88	1.17	1.13	1.05
Osteoporotic bone fractures	131 (26)	0.91	1.05	1.02	1.05
Degenerative joint disease‡	113 (22)	1.20	1.18	1.11†	1.07
Diabetes	73 (14)	1.01	1.09	1.04	1.07†
Asthma	50 (10)	1.28	1.11	1.05	1.05

*Values close to 1.0 indicate that the measurement or test is not affected by the comorbidity.
†Nominal $P<0.05$ adjusted for age and sex; when adjusted for multiple comparisons, none was statistically significant.
‡Osteoarthritis and degenerative joint disease were listed as separate conditions on the case report forms.
doi:10.1371/journal.pone.0060635.t005

acceptable performance were carried forward into clinical studies and ultimately, algorithm development.

Markers were evaluated based on relationship to disease activity and contribution to models thereof in the serial biomarker prioritization studies. Furthermore, both univariate and multivariate statistical analyses of biomarker-based disease activity prediction were used for biomarker prioritization, so as not to miss biomarkers that add value in multivariate models despite weaker individual relationships. Multiple statistical modeling methodologies were also applied in order to identify those that performed best on this problem. The ranking of biomarkers was based on both univariate and multivariate analyses and on a range of disease activity metrics, in order to reduce unintentional elimination of potentially useful biomarkers. Assessment in multiple, sequential studies was used to reduce the overall false discovery rate and to test the robustness of findings across studies.

Although relationships between biomarkers and various disease activity measures were assessed during the development process, the DAS28-CRP was chosen as the reference for final algorithm training because of its established validation against the DAS28-ESR [6], a disease activity measure shown to predict RA outcomes in clinical trials [49], and because measurement of CRP is more readily standardized than ESR in banked samples from multiple studies and sites. We found that multi-biomarker based models outperformed individual biomarkers at prediction of DAS28-CRP. Pilot imaging analyses indicated that disease activity scores from multivariate models trained to predict DAS28-CRP were also associated with synovitis and vascularity, assessed by power Doppler ultrasound, and with radiographically-determined joint

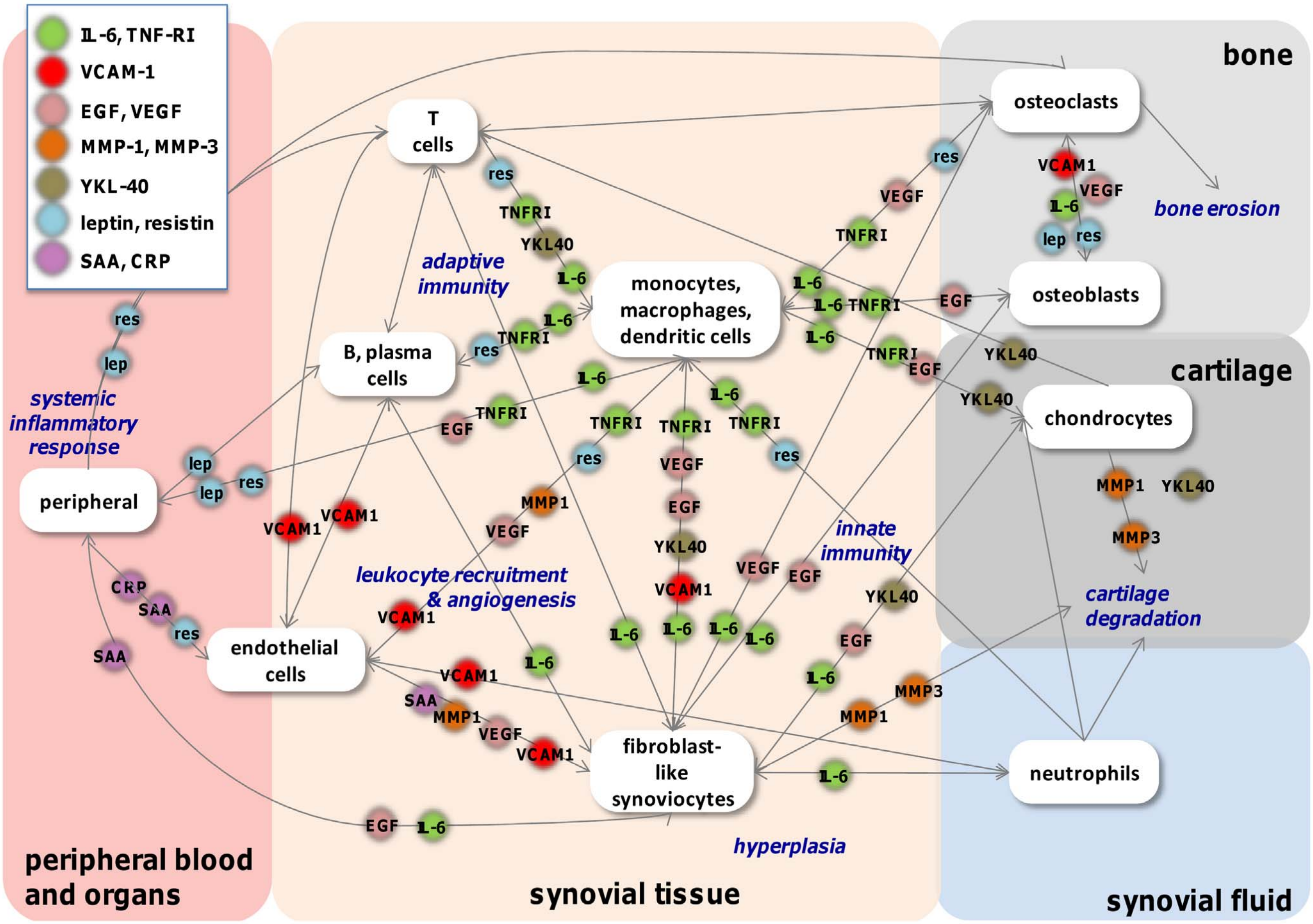

Figure 4. Network map of MBDA biomarker roles in cellular communication in RA.
doi:10.1371/journal.pone.0060635.g004

damage progression. These results demonstrate that a multi-biomarker algorithm trained specifically against the DAS28-CRP can nonetheless provide information more broadly on disease activity and, critically, on disease outcomes. Clinically assessed disease activity measures exhibit significant inter-assessor and inter-subject variability [10,13], whereas the median coefficient of variation of the MBDA score in repeat runs of the same sample is <2% [44]. Clinical measures, while clearly valuable, are nonetheless imperfect standards for comparison, and independent measures such as ultrasound and radiographic imaging are important indicators of the activity of the fundamental disease processes.

Because comorbidities can conceivably influence clinical assessment and biomarker levels, we assessed the impact of several common comorbidities on the MBDA score and observed no confounding effects. Larger studies are needed to confirm and further define these observations. It is especially important to examine other comorbidities with inflammatory components that could not be analyzed in our data, such as infections and malignancies. Infections in particular are associated with rapid elevation of cytokines and acute phase reactants, so MBDA score results should be interpreted with caution in patients with active infections. In addition, the effects of vaccination merit examination since it can cause acute inflammation.

Although they were selected primarily via a statistics-driven process, the prioritized biomarkers reflect key biological pathways, cells, and features of RA. Figure 4 summarizes known roles of the 12 biomarkers included in the final algorithm in cellular interactions and processes important to the disease. The diagram illustrates a critical premise driving this multi-biomarker development effort: that a biologically diverse set of quantitative biomarkers might be able to provide more information about underlying disease biology than any single biomarker.

Despite the scope and rigor of the work described, some important limitations should be noted. For practical considerations, we restricted our evaluation to serum biomarkers that were measurable in RA patient serum using commercially available assays. As a result, cytokines and mediators that may be highly expressed within the rheumatoid joint, but that are difficult to detect in serum either due to low levels or inadequate assays, were not considered. In addition, the clinical studies that formed the basis for this work excluded some patients for a combination of ethical and practical reasons (e.g. around steroid use, pregnancy and serious medical condition). As a consequence, this development program cannot be considered to establish the applicability of the biomarker findings and MBDA test to these patient types, and further studies are required. Finally, the MBDA test is by design an exclusively biomarker-based assessment. While this allows for certain advantages, including efficiency and objectivity, it does not include physical examination and should not be seen as a replacement for clinician or patient assessment, but rather as an additional, complementary tool to provide objective, quantitative data to inform clinical decision-making.

The MBDA algorithm developed as described here has subsequently been evaluated and validated in independent cohorts [21,22]. Additional studies are underway to further evaluate the relationship between the MBDA score and other measures of disease activity, and the ability of the score to indicate risk of critical patient outcomes such as joint damage progression. Ultimately, prospective studies incorporating the MBDA score will be required to fully elucidate its clinical utility and its appropriate use alongside other approaches to patient assessment in routine practice.

Supporting Information

Table S1 Candidate biomarkers and assays used.
(XLS)

Table S2 Details of Assays used in Feasibility studies. All summary statistics are presented as the mean values across study plates. 'sample dilution' is the dilution of serum added to the assay after any optimization. 'avg sample CV' is mean % coefficient of variation, calculated using duplicate measurements of all samples on each plate. The Lower Measurability Limit (LML) is the standard of lowest concentration for which all replicates have higher results than all replicates of the blank and all standards of lower concentrations. Successive standards above the LML are eligible to represent the Higher Measurability Limit (HML) so long as all their replicates have higher results than all replicates for standards of lower concentration. The HML is the highest of these eligible standards for which all replicates have lower results than all standards of higher concentrations.
(XLS)

Table S3 Details of assays used in Development studies.
(XLS)

Table S4 Results for individual biomarkers in Feasibility (Stage 2) studies.
(XLS)

Author Contributions

Conceived and designed the experiments: MC GC MT CS DS DC LH J. Carulli PT NS MW J. Curtis. Performed the experiments: MC MT CS DS PT J. Curtis MW KS. Analyzed the data: MC GC YS SR NK DH PT KS. Wrote the paper: MC GC YS SR NK MT CS DS DH DC LH J. Carulli PT NS MW J. Curtis KS.

References

1. Bakker MF, Jacobs JW, Verstappen SM, Bijlsma JW (2007) Tight control in the treatment of rheumatoid arthritis: efficacy and feasibility. Ann Rheum Dis 66 Suppl 3: iii56–60. Available:http://www.ncbi.nlm.nih.gov/entrez/query.fcgi?cmd = Retrieve&db = PubMed&dopt = Citation&list_uids = 17934098.
2. Verstappen SMM, Jacobs JWG, van der Veen MJ, Heurkens AH, Schenk Y, et al. (2007) Intensive treatment with methotrexate in early rheumatoid arthritis: aiming for remission. Computer Assisted Management in Early Rheumatoid Arthritis (CAMERA, an open-label strategy trial). Annals of the rheumatic diseases 66: 1443–1449. Available:http://www.pubmedcentral.nih.gov/articlerender.fcgi?artid = 2111604&tool = pmcentrez&rendertype = abstract. Accessed 22 July 2011.
3. Mease PJ (2010) Improving the routine management of rheumatoid arthritis: the value of tight control. The Journal of rheumatology 37: 1570–1578. Available:http://www.ncbi.nlm.nih.gov/pubmed/20595287. Accessed 23 July 2012.
4. Smolen JS, Aletaha D, Bijlsma JWJ, Breedveld FC, Boumpas D, et al. (2010) Treating rheumatoid arthritis to target: recommendations of an international task force. Annals of the rheumatic diseases 69: 631–637. Available:http://www.pubmedcentral.nih.gov/articlerender.fcgi?artid = 3015099&tool = pmcentrez&rendertype = abstract. Accessed 24 June 2011.
5. Singh JA, Furst DE, Bharat A, Curtis JR, Kavanaugh AF, et al. (2012) 2012 update of the 2008 American College of Rheumatology recommendations for the use of disease-modifying antirheumatic drugs and biologic agents in the treatment of rheumatoid arthritis. Arthritis care & research 64: 625–639. Available:http://www.ncbi.nlm.nih.gov/pubmed/22473917. Accessed 15 July 2012.
6. Wells G, Becker J-C, Teng J, Dougados M, Schiff M, et al. (2009) Validation of the 28-joint Disease Activity Score (DAS28) and European League Against Rheumatism response criteria based on C-reactive protein against disease progression in patients with rheumatoid arthritis, and comparison with the DAS28 based on erythrocyte sedimentation rate. Annals of the rheumatic

diseases 68: 954–960. Available:http://www.pubmedcentral.nih.gov/articlerender.fcgi?artid = 2674547&tool = pmcentrez&rendertype = abstract. Accessed 18 July 2011.

7. Fransen J, van Riel PLCM (n.d.) The Disease Activity Score and the EULAR response criteria. Clinical and experimental rheumatology 23: S93–9. Available:http://www.ncbi.nlm.nih.gov/pubmed/16273792. Accessed 22 October 2012.
8. Zatarain E, Strand V (2006) Monitoring disease activity of rheumatoid arthritis in clinical practice: contributions from clinical trials. Nature clinical practice Rheumatology 2: 611–618. Available:http://www.ncbi.nlm.nih.gov/pubmed/17075600. Accessed 25 January 2012.
9. Pincus T, Swearingen CJ, Bergman M, Yazici Y (2008) RAPID3 (Routine Assessment of Patient Index Data 3), a rheumatoid arthritis index without formal joint counts for routine care: proposed severity categories compared to disease activity score and clinical disease activity index categories. The Journal of rheumatology 35: 2136–2147. Available:http://www.ncbi.nlm.nih.gov/pubmed/18793006. Accessed 7 November 2012.
10. Uhlig T, Kvien TK, Pincus T (2009) Test-retest reliability of disease activity core set measures and indices in rheumatoid arthritis. Annals of the rheumatic diseases 68: 972–975. Available:http://www.ncbi.nlm.nih.gov/pubmed/18957489. Accessed 22 July 2011.
11. Ranzolin A, Brenol JCT, Bredemeier M, Guarienti J, Rizzatti M, et al. (2009) Association of concomitant fibromyalgia with worse disease activity score in 28 joints, health assessment questionnaire, and short form 36 scores in patients with rheumatoid arthritis. Arthritis and rheumatism 61: 794–800. Available:http://www.ncbi.nlm.nih.gov/pubmed/19479706. Accessed 23 August 2011.
12. Leeb BF, Andel I, Sautner J, Nothnagl T, Rintelen B (2004) The DAS28 in rheumatoid arthritis and fibromyalgia patients. Rheumatology (Oxford, England) 43: 1504–1507. Available:http://www.ncbi.nlm.nih.gov/pubmed/15252215. Accessed 25 January 2012.
13. Marhadour T, Jousse-Joulin S, Chalès G, Grange L, Hacquard C, et al. (2010) Reproducibility of joint swelling assessments in long-lasting rheumatoid arthritis: influence on Disease Activity Score-28 values (SEA-Repro study part I). The Journal of rheumatology 37: 932–937. Available:http://www.ncbi.nlm.nih.gov/pubmed/20360184. Accessed 25 January 2012.
14. Sokka T, Pincus T (2009) Erythrocyte sedimentation rate, C-reactive protein, or rheumatoid factor are normal at presentation in 35%–45% of patients with rheumatoid arthritis seen between 1980 and 2004: analyses from Finland and the United States. J Rheumatol 36: 1387–1390. Available:http://www.ncbi.nlm.nih.gov/entrez/query.fcgi?cmd = Retrieve&db = PubMed&dopt = Citation&list_uids = 19411389.
15. Rhodes B, Merriman ME, Harrison A, Nissen MJ, Smith M, et al. (2010) A genetic association study of serum acute-phase C-reactive protein levels in rheumatoid arthritis: implications for clinical interpretation. PLoS Med 7: e1000341. Available:http://www.ncbi.nlm.nih.gov/entrez/query.fcgi?cmd = Retrieve&db = PubMed&dopt = Citation&list_uids = 20877716.
16. Keenan RT, Swearingen CJ, Yazici Y (2008) Erythrocyte sedimentation rate and C-reactive protein levels are poorly correlated with clinical measures of disease activity in rheumatoid arthritis, systemic lupus erythematosus and osteoarthritis patients. Clin Exp Rheumatol 26: 814–819. Available:http://www.ncbi.nlm.nih.gov/entrez/query.fcgi?cmd = Retrieve&db = PubMed&dopt = Citation&list_uids = 19032813.
17. Mease PJ (1998) The potential roles for novel biomarkers in rheumatoid arthritis assessment. Clinical and experimental rheumatology 29: 567–574. Available:http://www.ncbi.nlm.nih.gov/pubmed/21640052. Accessed 18 July 2012.
18. Chambers RE, MacFarlane DG, Whicher JT, Dieppe P a (1983) Serum amyloid-A protein concentration in rheumatoid arthritis and its role in monitoring disease activity. Annals of the rheumatic diseases 42: 665–667. Available:http://www.pubmedcentral.nih.gov/articlerender.fcgi?artid = 1001325&tool = pmcentrez&rendertype = abstract. Accessed 18 July 2012.
19. Klimiuk P a, Sierakowski S, Latosiewicz R, Cylwik JP, Cylwik B, et al. (2002) Soluble adhesion molecules (ICAM-1, VCAM-1, and E-selectin) and vascular endothelial growth factor (VEGF) in patients with distinct variants of rheumatoid synovitis. Annals of the rheumatic diseases 61: 804–809. Available:http://www.pubmedcentral.nih.gov/articlerender.fcgi?artid = 1754213&tool = pmcentrez&rendertype = abstract.
20. Hammer HB, Odegard S, Fagerhol MK, Landewé R, van der Heijde D, et al. (2007) Calprotectin (a major leucocyte protein) is strongly and independently correlated with joint inflammation and damage in rheumatoid arthritis. Annals of the rheumatic diseases 66: 1093–1097. Available:http://www.pubmedcentral.nih.gov/articlerender.fcgi?artid = 1954700&tool = pmcentrez&rendertype = abstract. Accessed 18 July 2012.
21. Bakker M, Cavet G, Jacobs J, Bijlsma J, Haney D, et al. (2012) Performance of a Multi-Biomarker Score Measuring Rheumatoid Arthritis Disease Activity in the CAMERA Tight Control Study. Annals of Rheumatic Disease.
22. Curtis JR, van der Helm-van Mil AH, Knevel R, Huizinga TW, Haney DJ, et al. (2012) Validation of a novel multi-biomarker test to assess rheumatoid arthritis disease activity. Arthritis care & research. Available:http://www.ncbi.nlm.nih.gov/pubmed/22736476. Accessed 22 July 2012.
23. Arnett FC, Edworthy SM, Bloch DA, McShane DJ, Fries JF, et al. (1988) The American Rheumatism Association 1987 revised criteria for the classification of rheumatoid arthritis. Arthritis and rheumatism 31: 315–324. Available:http://www.ncbi.nlm.nih.gov/pubmed/3358796. Accessed 18 July 2012.
24. Iannaccone CK, Lee YC, Cui J, Frits ML, Glass RJ, et al. (2011) Using genetic and clinical data to understand response to disease-modifying anti-rheumatic drug therapy: data from the Brigham and Women's Hospital Rheumatoid Arthritis Sequential Study. Rheumatology (Oxford, England) 50: 40–46. Available:http://www.ncbi.nlm.nih.gov/pubmed/20847201. Accessed 18 July 2012.
25. Karlson EW, Chibnik LB, Cui J, Plenge RM, Glass RJ, et al. (2008) Associations between human leukocyte antigen, PTPN22, CTLA4 genotypes and rheumatoid arthritis phenotypes of autoantibody status, age at diagnosis and erosions in a large cohort study. Annals of the rheumatic diseases 67: 358–363. Available:http://www.pubmedcentral.nih.gov/articlerender.fcgi?artid = 2945890&tool = pmcentrez&rendertype = abstract. Accessed 18 July 2012.
26. Taylor PC, Steuer A, Gruber J, Cosgrove DO, Blomley MJK, et al. (2004) Comparison of ultrasonographic assessment of synovitis and joint vascularity with radiographic evaluation in a randomized, placebo-controlled study of infliximab therapy in early rheumatoid arthritis. Arthritis and rheumatism 50: 1107–1116. Available:http://www.ncbi.nlm.nih.gov/pubmed/15077292. Accessed 18 July 2012.
27. Taylor PC, Steuer A, Gruber J, McClinton C, Cosgrove DO, et al. (2006) Ultrasonographic and radiographic results from a two-year controlled trial of immediate or one-year-delayed addition of infliximab to ongoing methotrexate therapy in patients with erosive early rheumatoid arthritis. Arthritis and rheumatism 54: 47–53. Available:http://www.ncbi.nlm.nih.gov/pubmed/16385521. Accessed 18 July 2012.
28. Fleischmann RM, Curtis JR, Hamburger MH, Blumstein H, Swan K, et al. (2010) RA population characteristics in InFoRM, a longitudinal observational study. Ann Rheum Dis 69: 657.
29. Wren JD, Garner HR (2004) Shared relationship analysis: ranking set cohesion and commonalities within a literature-derived relationship network. Bioinformatics (Oxford, England) 20: 191–198. Available:http://bioinformatics.oxfordjournals.org/cgi/doi/10.1093/bioinformatics/btg390. Accessed 18 July 2012.
30. Eastman PS, Manning WC, Qureshi F, Haney D, Cavet G, et al. (2012) Characterization of a multiplex, 12-biomarker test for rheumatoid arthritis. Journal of pharmaceutical and biomedical analysis 70: 415–424. Available:http://www.ncbi.nlm.nih.gov/pubmed/22749821. Accessed 22 October 2012.
31. Todd DJ, Knowlton N, Amato M, Frank MB, Schur PH, et al. (2011) Erroneous augmentation of multiplex assay measurements in patients with rheumatoid arthritis due to heterophilic binding by serum rheumatoid factor. Arthritis and rheumatism 63: 894–903. Available:http://www.ncbi.nlm.nih.gov/pubmed/21305505. Accessed 18 July 2012.
32. Benjamini Y, Hochberg M (1995) Controlling the false discovery rate: a practical and powerful approach to multiple testing. Journal of the Royal Statistical Society: Series B (Statistical Methodology) 57: 289–300. Available:http://www.jstor.org/stable/10.2307/2346101. Accessed 19 July 2012.
33. Tibshirani R (1996) Regression shrinkage and selection via the lasso. Journal of the Royal Statistical Society: Series B (Statistical Methodology) 58: 267–288. Available:http://www.jstor.org/stable/10.2307/2346178. Accessed 19 July 2012.
34. Zou H, Hastie T (2005) Regularization and variable selection via the elastic net. Journal of the Royal Statistical Society: Series B (Statistical Methodology) 67: 301–320. Available:http://doi.wiley.com/10.1111/j.1467-9868.2005.00503.x.
35. Hastie T, Tibshirani R, Friedman J (2009) The Elements of Statistical Learning: Data Mining, Inference, and Prediction. New York: Springer. 746 p. Available:http://www.springer.com/statistics/statistical+theory+and+methods/book/978-0-387-84857-0. Accessed 22 July 2012.
36. Breiman L, Friedman JH (1997) Predicting Multivariate Responses in Multiple Linear Regression. Journal of the Royal Statistical Society: Series B (Statistical Methodology) 59: 3–54. Available:http://onlinelibrary.wiley.com/doi/10.1111/1467-9868.00054/full. Accessed 19 July 2012.
37. Targońska-Stepniak B, Majdan M, Dryglewska M (2008) Leptin serum levels in rheumatoid arthritis patients: relation to disease duration and activity. Rheumatology international 28: 585–591. Available:http://www.ncbi.nlm.nih.gov/pubmed/17968549. Accessed 18 July 2012.
38. Rioja I, Hughes FJ, Sharp CH, Warnock LC, Montgomery DS, et al. (2008) Potential novel biomarkers of disease activity in rheumatoid arthritis patients: CXCL13, CCL23, transforming growth factor alpha, tumor necrosis factor receptor superfamily member 9, and macrophage colony-stimulating factor. Arthritis and rheumatism 58: 2257–2267. Available:http://www.ncbi.nlm.nih.gov/pubmed/18668547. Accessed 18 July 2012.
39. Melis L, Vandooren B, Kruithof E, Jacques P, De Vos M, et al. (2010) Systemic levels of IL-23 are strongly associated with disease activity in rheumatoid arthritis but not spondyloarthritis. Annals of the rheumatic diseases 69: 618–623. Available:http://www.ncbi.nlm.nih.gov/pubmed/19196728. Accessed 19 July 2012.
40. Park M-C, Chung SJ, Jung S-J, Park Y-B, Lee S-K (2004) Relationship of serum TWEAK level to cytokine level, disease activity, and response to anti-TNF treatment in patients with rheumatoid arthritis. Scandinavian journal of rheumatology 37: 173–178. Available:http://www.ncbi.nlm.nih.gov/pubmed/18465450. Accessed 19 July 2012.
41. Ehrchen JM, Sunderkötter C, Foell D, Vogl T, Roth J (2009) The endogenous Toll-like receptor 4 agonist S100A8/S100A9 (calprotectin) as innate amplifier of infection, autoimmunity, and cancer. Journal of leukocyte biology 86: 557–566.

Available:http://www.ncbi.nlm.nih.gov/pubmed/19451397. Accessed 19 July 2012.

42. Inoue E, Yamanaka H, Hara M, Tomatsu T, Kamatani N (2007) Comparison of Disease Activity Score (DAS)28- erythrocyte sedimentation rate and DAS28-C-reactive protein threshold values. Annals of the rheumatic diseases 66: 407–409. Available:http://www.pubmedcentral.nih.gov/articlerender.fcgi?artid = 1856019&tool = pmcentrez&rendertype = abstract. Accessed 25 January 2012.
43. Lavie F, Miceli-Richard C, Ittah M, Sellam J, Gottenberg J-E, et al. (2007) Increase of B cell-activating factor of the TNF family (BAFF) after rituximab treatment: insights into a new regulating system of BAFF production. Annals of the rheumatic diseases 66: 700–703. Available:http://www.pubmedcentral.nih.gov/articlerender.fcgi?artid = 1954605&tool = pmcentrez&rendertype = abstract. Accessed 19 July 2012.
44. Eastman PS, Manning WC, Qureshi F, Haney D, Cavet G, et al. (2012) Characterization of a multiplex, 12-biomarker test for rheumatoid arthritis. Journal of pharmaceutical and biomedical analysis. Available:http://www.ncbi.nlm.nih.gov/pubmed/22749821. Accessed 22 July 2012.
45. Inoue E, Yamanaka H, Hara M, Tomatsu T, Kamatani N (2007) Comparison of Disease Activity Score (DAS)28- erythrocyte sedimentation rate and DAS28-C-reactive protein threshold values. Annals of the rheumatic diseases 66: 407–409. Available:http://www.pubmedcentral.nih.gov/articlerender.fcgi?artid = 1856019&tool = pmcentrez&rendertype = abstract. Accessed 25 January 2012.
46. Paik S, Tang G, Shak S, Kim C, Baker J, et al. (2006) Gene expression and benefit of chemotherapy in women with node-negative, estrogen receptor-positive breast cancer. Journal of clinical oncology?: official journal of the American Society of Clinical Oncology 24: 3726–3734. Available:http://www.ncbi.nlm.nih.gov/pubmed/16720680. Accessed 13 July 2012.
47. Paik S, Shak S, Tang G, Kim C, Baker J, et al. (2004) A multigene assay to predict recurrence of tamoxifen-treated, node-negative breast cancer. The New England journal of medicine 351: 2817–2826. Available:http://www.ncbi.nlm.nih.gov/pubmed/15591335.
48. Kolberg JA, Jørgensen T, Gerwien RW, Hamren S, McKenna MP, et al. (2009) Development of a type 2 diabetes risk model from a panel of serum biomarkers from the Inter99 cohort. Diabetes care 32: 1207–1212. Available:http://www.pubmedcentral.nih.gov/articlerender.fcgi?artid = 2699726&tool = pmcentrez&rendertype = abstract. Accessed 13 July 2012.
49. Prevoo ML, van't Hof MA, Kuper HH, van Leeuwen MA, van de Putte LB, et al. (1995) Modified disease activity scores that include twenty-eight-joint counts. Development and validation in a prospective longitudinal study of patients with rheumatoid arthritis. Arthritis Rheum 38: 44–48. Available:http://www.ncbi.nlm.nih.gov/entrez/query.fcgi?cmd = Retrieve&db = PubMed&dopt = Citation&list_uids = 7818570.
